# Wolf in sheep’s clothes: An uncommon case of pneumonic-type adenocarcinoma

**DOI:** 10.7196/AJTCCM.2021.v27i1.048

**Published:** 2021-03-09

**Authors:** S D Maasdorp

**Affiliations:** Division of Pulmonology and Critical Care, Departments of Internal Medicine and Surgery, Faculty of Health Sciences, University of the Free State, Bloemfontein, South Africa

**Keywords:** lung cancer, adenocarcinoma, bronchioloalveolar carcinoma, non-resolving pneumonia

## Abstract

We report a case of a patient who presented with clinical and radiological features of a non-resolving pneumonia. Special investigations and a poor response to antibiotic therapy excluded an infective aetiology. A diagnosis of invasive mucinous adenocarcinoma, previously termed bronchioloalveolar carcinoma, was made from lung biopsy. This case illustrates the challenges of establishing a timely diagnosis of an uncommon pneumonic-type of adenocarcinoma.

## Case

A 55-year-old female was referred to the pulmonology outpatient department with a history of a persistent productive cough of ‘pink’ sputum for the preceding 12 months. During this time, she also developed progressively worsening dyspnoea and had grade 3 dyspnoea on the modified Medical Research Council scale at the time of presentation to the clinic. No other respiratory symptoms such as wheezing or chest pain were reported and she did not have any constitutional symptoms of fever, night sweats or weight loss. She had no history of any childhood respiratory illnesses nor did she have any history of previous tuberculosis. The patient never smoked and maintained sober habits. Her family history was non-contributory, and she was employed as a domestic worker with no significant exposures that she could recall. She had hypertension and was on chronic medication that included spironolactone, furosemide, amlodipine, aspirin, simvastatin and oestrogen replacement therapy. She previously underwent hysterectomy for abnormal uterine bleeding and no malignancy was detected at the time.

She was treated with a 7-day course of amoxicillin-clavulanic acid for supposed community-acquired pneumonia at a local hospital prior to referral. Sputum for microscopy, culture and sensitivity during this time did not yield any pathogens. The result of a tuberculosis GeneXpert test was negative, but *Mycobacterium tuberculosis* culture or investigations for atypical pathogens were not done. Blood tests showed a white cell count of 7.3 × 10^9^ /L, haemoglobin level of 13.6 g/dL, platelet count of 267 × 10^9^ /L, C-reactive protein level of 21 mg/L and she tested negative for HIV. Although a chest radiograph performed at the local hospital was described as revealing ‘extensive opacification of the left lung’, the image was not presented at the clinic and thus could not be reviewed. In view of her poor response to treatment and blood results that were not in keeping with infection, she was referred to us for further evaluation

**Fig. 1:**
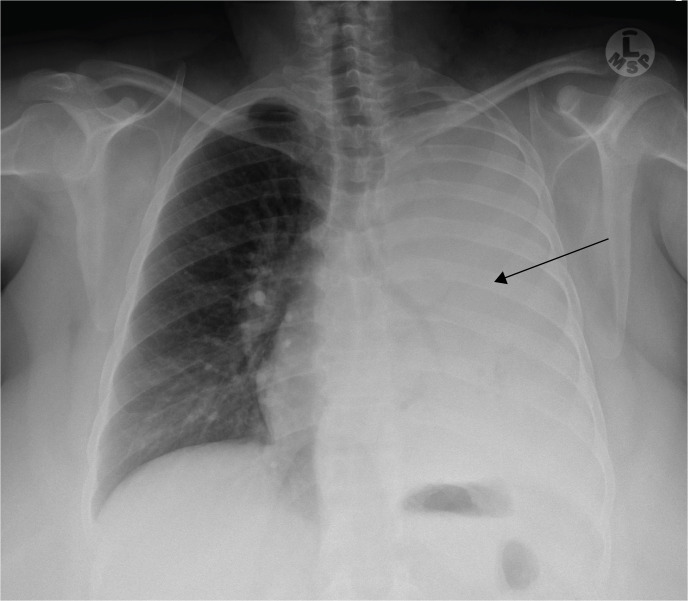
Chest X-ray image showing complete opacification of the left lung.

**Fig. 2:**
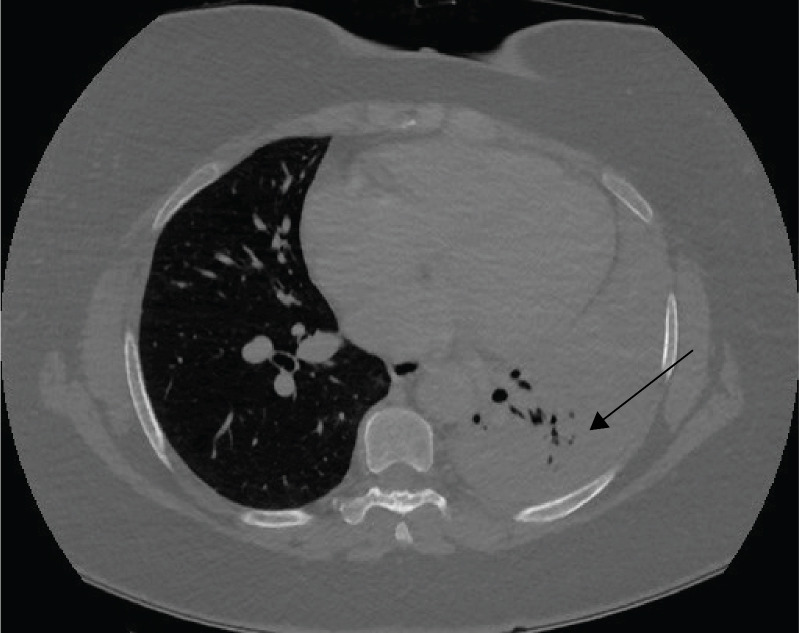
Chest computed tomography image confirming consolidation of the left lung

On clinical examination, she was obese with a body mass index of 48.24 kg/m² . Her blood pressure was 120/80 mmHg, pulse rate was 72 beats per minute (bpm), respiratory rate was 15 breaths per minute and oxygen saturation was 93% measured by pulse oximetry while breathing room air at rest. Arterial blood gas testing was not performed. On respiratory examination, her trachea was centrally situated, but she had dullness to percussion and bronchial breathing of the whole of the left hemithorax, while she had normal percussion and vesicular breath sounds on the right. The rest of the clinical examination was within normal limits. A chest X-ray image at the time of initial assessment revealed complete opacification of the left lung with associated air bronchogrammes noted in the left perihilar region (Fig. 1). Chest computed tomography analysis confirmed dense consolidation of the left lung with no evidence of atelectasis or pleural effusion (Fig. 2).

**Fig. 3:**
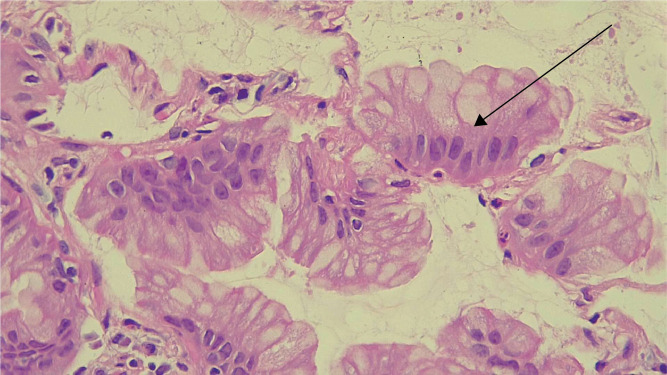
Intermediate magnification micrograph of lung biopsy histology revealing lepidic growth pattern of neoplastic cells after haematoxylin and eosin staining

In view of the clinical problem of a non-resolving pneumonia, the differential diagnoses included atypical pneumonia, non-tuberculous mycobacterial disease, primary lymphoma of the lung and slow-growing primary lung neoplasms. Flexible bronchoscopy was therefore performed to obtain respiratory specimens for further analysis. Inspection of the tracheobronchial tree revealed an excessive amount of watery sputum in the airways in keeping with bronchorrhea. Bronchoalveolar lavage specimens from the left upper lobe were sent for microscopy, culture and polymerase chain reaction (PCR) tests for *Mycobacterium tuberculosis, Pneumocystis jirovecii, Mycoplasma pneumoniae* and *Chlamydia pneumoniae*. All PCR tests were negative. Flow cytometry of bronchoalveolar lavage fluid found a normal ratio of CD4:CD8 cells, in keeping with a reactive T-cell response. Transbronchial lung biopsies, however, revealed tumour tissue with the appearance of a well-differentiated mucus-secreting adenocarcinoma with lepidic features (Fig. 3).

Immunohistochemical staining for cytokeratin 7 (CK7), thyroid transcription factor-1 (TTF-1) and napsin A were positive, while staining for CK20 was negative. A final diagnosis of invasive mucinous adenocarcinoma (previously known as bronchioloalveolar carcinoma) was therefore established.

## Discussion

In 2018, there were ~2.1 million new cases and ~1.8 million deaths due to lung cancer, making lung cancer the leading cause of cancer-related deaths worldwide.^[1]^ Smoking is the most important risk factor associated with lung cancer, but other factors such as second-hand smoke inhalation, ionising radiation, occupational exposure to environmental toxins such as asbestos, silica and polycyclic aromatic hydrocarbons as well as a positive family history, are also associated with an increased risk for developing lung cancer.^[2]^ The World Health Organization (WHO) classified epithelial tumours of the lung into various types such as adenocarcinoma, squamous cell carcinoma and neuroendocrine tumours, with variants of each type described based on the predominant cell type, growth pattern and extent of invasion.^[3]^ Apart from exhibiting an acinar/tubular structure or producing mucin, adenocarcinoma of the lung is also characterised immunohistochemically by TTF-1 and/or napsin A positivity, and frequently harbours driver mutations in genes such as epidermal growth factor receptor (EGFR), anaplastic lymphoma kinase (ALK), c-ros oncogene 1 (ROS1) and rearranged during transfection (RET), for which targeted molecular therapies have proven to be valuable treatment modalities.^[4]^

Historically, Musser described a diffuse infiltrative type of lung cancer that involved either a single lobe or an entire lung in 1903.^[5]^ It was later noted that the neoplastic cells actually grew along alveolar walls in a characteristic lepidic pattern and in 1960, Avril Liebow coined the term ‘bronchioloalveolar carcinoma’ to describe this type of lung tumour.^[6]^ In 2015, the WHO changed the histological classification of lung cancer and the term ‘bronchioloalveolar carcinoma’ became redundant.^[4]^ The underlying histology of the previously termed ‘bronchioloalveolar carcinoma’ usually represents a non-invasive or minimally invasive adenocarcinoma of the lung. The histological features can however be quite variable. Sub-types of adenocarcinoma such as invasive adenocarcinoma, minimally invasive adenocarcinoma and pre-invasive lesions such as atypical adenomatous hyperplasia and adenocarcinoma *in situ*^[4]^ could have been previously diagnosed as ‘bronchioloalveolar carcinoma’ of the lung. Various radiological presentations of adenocarcinoma such as solid, bubbling or pneumonic-type of lung cancer are also described.^[5]^ The pneumonic-type of lung cancer, as is described in the current case report, is usually an invasive mucinous adenocarcinoma and occurs in adults between the ages of 41 and 66 years, with no gender preponderance.^[5]^ Consolidation occurs in 83% of cases, but the tumour can involve multiple lobes of the lungs.^[5]^ Nodal or systemic metastases are rare.^[5]^ In view of the overlapping symptoms such as cough, sputum production, dyspnoea, weight loss, haemoptysis, fever, bronchorrhea,^[7]^ and radiological features, this type of adenocarcinoma of the lung is often misdiagnosed as pneumonia, leading to a substantial delay in definitive management.^[8]^ Adenocarcinoma of the lung has a 5-year survival rate of 76 - 100%, although the prognosis of the pneumonic-type of lung cancer is worse than that of the bubbling or solid type.^[9]^ Our patient successfully underwent left-sided pneumonectomy and was coping well with cisplatin and gemcitabine chemotherapy at the time of writing this report.
